# Clinical significance of the histopathological metastatic largest lymph node size in colorectal cancer patients

**DOI:** 10.3389/fonc.2023.1120753

**Published:** 2023-03-06

**Authors:** Sinan Omeroglu, Selcuk Gulmez, Orhan Uzun, Aziz Serkan Senger, Ozgur Bostanci, Onur Guven, Erdal Polat, Mustafa Duman

**Affiliations:** ^1^ Department of General Surgery, University of Health Sciences Sisli Hamidiye Etfal Research and Training Hospital, Istanbul, Türkiye; ^2^ Department of Gastrointestinal Surgery, University of Health Sciences Kosuyolu High Specialization Education and Research Hospital, Istanbul, Türkiye

**Keywords:** colorectal cancer, lymph node metastasis, lymph node size, survival, postoperative complications

## Abstract

**Background:**

The metastatic lymph nodes (MLN) are interpreted to be correlated with prognosis of the colorectal cancers (CRC). The present retrospective study aimed to investigate the clinical significance of the largest MLN size in terms of postoperative outcomes and its predictive value in the prognosis of the patients with stage III CRC.

**Methods:**

Between May 2013 and December 2018, a total of 101 patients who underwent curative resection for stage III CRC retrospectively reviewed. All patients were divided into two groups regarding cut-off value (<1.05 cm and ≥1.05 cm) of maximum MLN diameter measured histopathologically. A comparative analysis of demographic and clinicopathological characteristics of the patients and their postoperative outcomes were performed.

**Results:**

Two groups carried similar demographic data and preoperative laboratory variables except the lymphocyte count, hematocrit (HCT) ratio, hemoglobin level and mean corpuscular volume (MCV) value (p<0.05). The patients with MLN diameter ≥1.05 cm (n=46) needed more erythrocyte suspension and were hospitalized longer than the patients with a diameter <1.05 cm (n=55) (p=0.006 and 0.0294, respectively). Patients with MLN diameter < 1.05 cm had a significantly longer overall survival than patients with MLN diameter ≥ 1.05 cm (75,29 *vs.* 52,57 months, respectively). Regarding the histopathologic features, the patients with MLN diameter ≥1.05 cm had larger tumor size and higher number of MLN than those with diameter <1.05 cm (p=0.049 and 0.001).

**Conclusion:**

The size of MLN larger than 1.05 cm may be predictive for a poor prognosis and lower survival of stage III CRC patients. The largest MLN size may be a proper alternative factor to the number of MLNs in predicting prognosis or in staging CRC patients.

## Introduction

Colorectal cancer (CRC) is the third leading cause of cancer-related death in the world, with an increasing incidence in developing countries (1). According to Globocan 2020 data, the number of new colorectal cancer cases in 2020 constituted 10% of all cancers and the number was 1,931,590 (1,065,960 males, 865,630 females). The cumulative incidence risk was 2.25% in both sexes, 2.71% in men and 1.83% in women. The number of deaths from colorectal cancer was 935,173 people and this rate was 9.4% of all cancer-related deaths (2). 5-year survival was detected as 65% among the colorectal cancer patients (3).

Surgery is the curative treatment of CRC. Following surgical resection, examination of lymph nodes (LNs) are important for staging, postoperative treatment approach, clinical follow-up and prognosis. LN metastasis plays an important role in the recurrence and survival of the CRC patients undergoing surgery (4). Total mesorectal or complete mesocolic excision and the number of metastatic LNs are well-known prognostic factors (5, 6). Also, the number of harvested LN and metastatic lymph node (MLN) ratio are important prognostic factors (7). Eighth Edition of The American Joint Committee on Cancer (AJCC) Staging Manual is currently used for pathological examination. In this TNM classification, N staging is done by the number of MLN, neither MLN size nor MLN ratio is considered. Similar to the LN rate, the effect of the size of the positive LN on the pathological stage is not being taken into account in this staging system (8).

There are studies in the literature evaluating the relationship between tumor size and CRC. Alese et al. reported that tumor size showed variable postoperative outcomes among CRC patients with the same AJCC stages. Therefore, they stated that tumor size may have a role in staging models for optimal management selection (9). Similarly, in some studies involving gastric and esophageal cancers, it was reported that MLN size was effective in determination of the prognosis and provided valuable support to the classification systems (10–12). There are limited reports describing the predictive value of MLN size on prognosis and survival in colorectal cancer (13, 14). The role of MLN size on postoperative outcomes remains a serious gap in the literature. Furthermore, to our knowledge, there is no research in the literature evaluating the relationship between metastatic largest LN size and postoperative complications in the patients with CRC. In the present retrospective study, we aimed to investigate the effect of the histopathologically determined metastatic largest LN size on postoperative outcomes in patients with stage III CRC.

## Materials and methods

### Study design and patient selection

**Figure 1:**
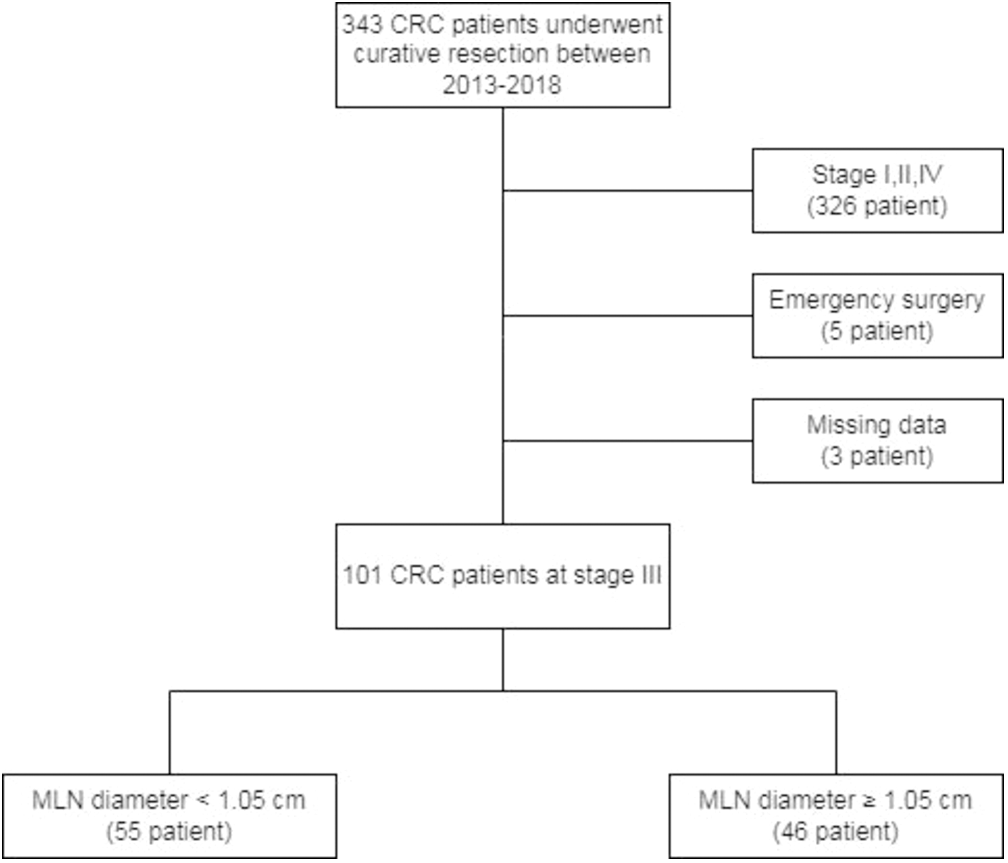
Flow chart of the study selection process.

Between May 2013 and December 2018, all patients who underwent curative surgery for CRC were retrospectively reviewed. Medical records of the patients who met inclusion criteria were collected. A total of 101 patients, aged ≥18 years who presented with stage III carcinoma of the colon or rectum histopathologically confirmed according to the TNM staging of the 8^th^ edition of AJCC Staging Manual and who underwent curative resection of the primary tumor enrolled in the study. Patients who underwent emergency operations or palliative resection, immunodeficiency patients or patients using immunomodulatory drugs, patients with the lymphoproliferative disease, patients with missing clinical or histopathological data were excluded from the study (**Figure 1**). The study was carried out in accordance with the Helsinki Declaration and local laws and regulations. This study was approved by the ethical committee of Koşuyolu High Specialization Education and Research Hospital (Date: 10th Nov 2020, Issue number: 2020/12/382).

### Data collection

Demographic data included age, sex, body mass index (BMI) and comorbidities defined by Charlson Comorbidity Index (CCI). The preoperative data included neoadjuvant therapy status, The American Society of Anesthesiologists (ASA) physical status score, localization of the tumor and laboratory data from whole blood analysis. Intraoperative data included the date of surgery, surgical technique, application of epidural anesthesia, duration of operation, and the need for erythrocyte suspension (ES) during the surgery. Postoperative data included length of hospital stay, the need for ES after an operation, Clavien-Dindo postoperative complication grade, date of the last follow-up and date of death. The pathological information included the histological type, maximum tumor diameter, tumor nuclear grade, total number of harvested LN, number of MLN, the diameter of largest MLN, the presence of lymphovascular invasion and perineural invasion.

Surgical specimens fixed with 10% buffered formalin for 24 hours were examined by the pathologist, and the long axis diameter of each dissected maximum MLN was measured and recorded. Two groups of patients were defined according to the cut-off value of maximum MLN diameter as MLN diameter < 1.05 cm and ≥1.05 cm, and all demographic features, preoperative and postoperative outcomes of patients were compared statistically.

### Statistical methods

All statistical analysis were performed with Statistical Package for the Social Sciences version 23 (SPSS 23). The frequencies and percentages were determined for categorical variables. The mean, standard deviation, median, minimum and maximum values were determined for continuous variables. The distribution of continuous variables was tested with the Kolmogorov Smirnov test. Chi-square analysis was used for comparison of the categorical variables. If appropriate, the categorical variables were compared with the Fisher-Freeman Halton Test. The Mann Whitney U test was used to compare two independent groups for the variables that did not fulfill the assumption of normal distribution, and the Student-t test was used for the variables with normal distribution. Clinicopathological variables affecting mortality were analyzed by Cox regression analysis by considering survival time. Analysis of ROC curve was used to measure the significance and cut-off value of maximum MLN diameter in predicting the mortality. The method of De Long et al. was used to compare the area under the ROC curves. The Kaplan-Meier method and the log-rank test were used to conduct the survival analyzes of the largest MLN size. p value of <0.05 was considered significant.

## Results

The mean age of patients was 59.82 ± 13.28 years and 59.41% of all patients were male. The mean BMI was 28.41 ± 2.51 kg/m². Most of the tumors were localized in rectum and sigmoid colon (33.66% and 26.7%, respectively). Patients receiving neoadjuvant therapy constituted 29.7% of all patients. Most of patients had a CCI score of 2 and 3 (29.7% and 25.74%, respectively). The ASA score of 49.6% of the patients was 2 (**Table 1**).

**Table 1:** Patient characteristics.

Characteristics/Findings		N = 101
Age (year), Mean ± SD		59.82±13.28
Sex, N (%)	Male	60 (59.41)
	Female	41 (40.59)
BMI (kg/m²), Mean ± SD		28.41±2.51
Localization of tumor, N (%)	Cecum	13 (12.87)
	Ascending colon	16 (15.84)
	Ascending colon+Sigmoid colon	1 (0.99)
	Transvers colon	3 (2.97)
	Descending colon	7 (6.93)
	Sigmoid colon	27 (26.7)
	Rectum	34 (33.66)
Neoadjuvant therapy, N (%)		30 (29.70)
CCI score, N (%)	0	16 (15.84)
	1	20 (19.80)
	2	30 (29.70)
	3	26 (25.74)
	4	7 (6.93)
	5	2 (1.98)
ASA score	1	23 (22.8)
	2	50 (49.6)
	3	28 (27.7)

SD, Standard deviation; BMI, Body mass index; CCI, Charlson Comorbidity Index; ASA, The American Society of Anesthesiologists.

**Figure 2:**
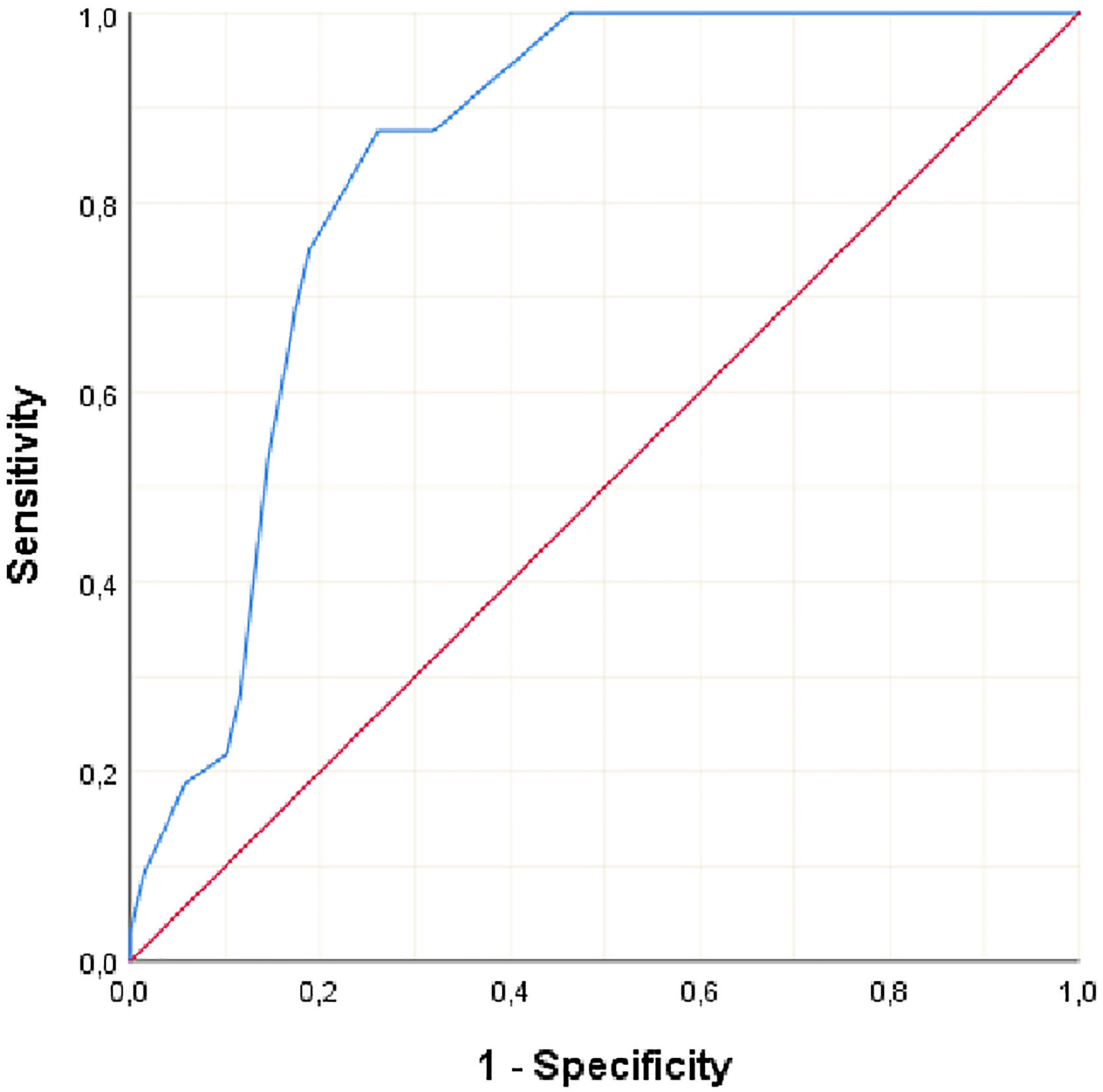
ROC analysis of maximum MLN diameter to predict mortality of the CRC patients.

**Figure 3:**
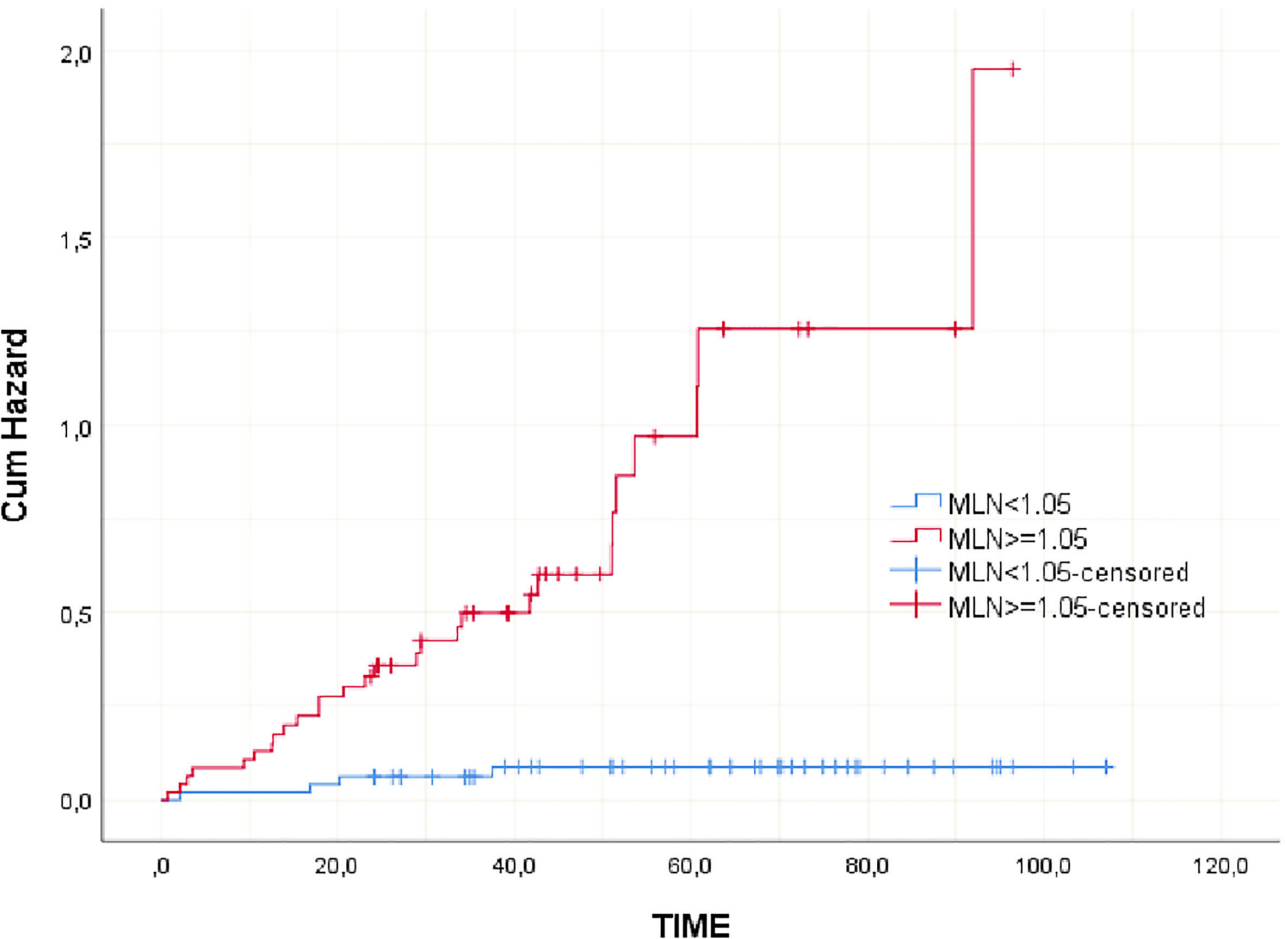
Graphs of cumulative hazard function for MLN categories.

Clinicopathological variables affecting mortality were evaluated by Cox regression analysis considering survival time and PNI, LVI, N stage, MLN size found significant (**Table 2**). The statistically significant cut-off value was 1.05 cm for the diameter of the largest MLN to predict mortality based on the ROC analysis (AUC: 0.841, p<0.001) (**Table 3**, **Figure 2**). According to this cut-off diameter of the largest MLN, all patients were divided into two groups as the MLN diameter < 1.05 cm and ≥ 1.05 cm. The relationship between MLN size and survival was evaluated by Kaplan-Meier analysis, and survival was found to be significantly higher in the group with MLN<1.05 cm compared to the group with MLN≥1.05 cm (**Table 4**, **Figure 3**) (p<0.001).

**Table 2:** Cox regression analysis of clinicopathological variables affecting mortality.

Parameter	Exp(B) (95,0% CI)	p
Tumor size	,977(,840;1,221)	,840
Sex	1,323(,562;3,400)	,562
Age	1,009(,563;1,039)	,563
LVI	1,681(1,393;2,702)	**,019**
PNI	1,588(,268;3,600)	**,021**
MLN size	2,428(1,139;3,556)	**<0,001**
N stage	2,292(,838;5,533)	**,011**
HGB	,882(,368;1,159)	,368
BMI	,877(,147;1,047)	,147

LVI, Lymphovascular invasion; PNI, Perineural invasion; MLN, Metastatic lymph node; HGB, Hemoglobin; BMI, Body mass index. P<0.05 statistically significant.

**Table 3:** ROC analysis of the maximum MLN diameter to predict mortality of the CRC patients.

	Diameter of maximum MLN
Cut-off	≥ 1.05 cm
AUC [LCI-UCI]	0.841 [0.765-0.917]
Sensitivity [LCI-UCI]	87.5 [71.0-96.5]
Specificity [LCI-UCI]	73.91 [61.9 – 83.7]
PPV	60.9
NPV	92.7
p value	**<0.001**

MLN, Metastatic lymph node.

LCI, 95% Lower Confidence Interval.

UCI, 95% Upper Confidence Interval. P<0.05 statistically significant.

**Table 4:** Kaplan-Meier analysis of the relationship between MLN size and survival.

Parameter	Category	Mean estimate (month) (95% CI)	P^a^
MLN size (cm)	MLN<1.05	75,297(68,039;80,596)	<0,001
	MLN≥1.05	52,570(44,254;60,686)	
	Overall survival	60,627(55,363;65,892)	

a Log Rank (Mantel-Cox); MLN, Metastatic lymph node.

There were 55 patients in the group with MLN diameter < 1.05 cm and 46 patients in the group with MLN diameter ≥ 1.05 cm. The age, sex, BMI, localization of tumor, neoadjuvant therapy status, CCI and ASA score did not differ significantly among two groups (**Table 5**). From the preoperative laboratory findings, lymphocyte count, hemoglobin level, HCT ratio were significantly higher and MCV value was lower in patients with MLN <1.05 cm compared to patients with MLN diameter ≥1.05 cm (p<0.05). Other laboratory findings did not differ among two groups (**Table 6**).

**Table 5:** Comparison of patient characteristics according to the diameter of largest MLN.

Characteristics/Findings		MLN < 1.05 cm (N = 55)	MLN ≥ 1.05 cm (N = 46)	P value
Age (year), Mean ± SD		59.47 ± 12.43	60.24 ± 14.37	0.566^a^
Sex, N (%)	Male	36 (65.5)	24 (52.2)	0.176^b^
	Female	19 (35.5)	22 (47.8)	
BMI (kg/m²), Mean ± SD		28.48 ± 2.73	28.33 ± 2.25	0.995^a^
Localization of tumor, N (%)	Cecum	8 (14.5)	5 (10.9)	0.432^b^
	Ascending colon	9 (16.4)	7 (15.2)	
	Ascending colon +sigmoid colon	1 (1.8)	0 (0)	
	Transvers colon	1 (1.8)	2 (4.4)	
	Descending colon	2 (3.6)	5 (10.9)	
	Sigmoid colon	18 (32.7)	9 (19.6)	
	Rectum	16 (29.1)	18 (39.1)	
Neoadjuvant therapy, N (%)		16 (29.1)	14 (30.4)	0.883^b^
CCI score, N (%)	0	8 (14.5)	8 (17.4)	0.713^b^
	1	11 (20)	9 (19.6)	
	2	17 (30.9)	13 (28.3)	
	3	16 (29.1)	10 (21.7)	
	4	3 (5.5)	4 (8.7)	
	5	0 (0)	2 (4.4)	
ASA score, N (%)	1	12 (21.8)	11 (23.9)	0.611^b^
	2	30 (54.6)	20 (43.5)	
	3	13 (23.6)	15 (32.6)	

^a^Student's t-test; ^b^Chi-Square test; MLN, Metastatic lymph node; SD, Standard deviation; BMI, Body mass index; CCI, Charlson Comorbidity Index; ASA, The American Society of Anesthesiologists.

**Table 6:** Preoperative laboratory findings.

Parameter	Total N = 101	MLN < 1.05 cm (N = 55)	MLN ≥ 1.05 cm (N = 46)	P value
CEA (µg/L)	4.3 [1.1-81.6]	4.5 [1.4-81.6]	4.25 [1.1-58.7]	0.203
CA 19-9 (kU/L)	13.1 [2.3-86.6]	12.3 [2.9-86.6]	14.3 [2.3-85.9]	0.943
WBC (10^9^/L)	7.1 [2.9-17.4]	6.7 [3.2-13.7]	7.15 [2.9-17.4]	0.881
Neutrophil (10^9^/L)	4.2 [1.4-13.5]	4.1 [1.7-9.8]	4.6 [1.4-13.5]	0.761
Lymphocyte (10^9^/L)	1.6 [0.5-4.3]	1.7 [0.7-4.3]	1.5 [0.5-3.4]	**0.014**
Monocyte (10^9^/L)	0.5 [0.1-1.7]	0.5 [0.1-1]	0.5 [0.3-1.7]	0.609
Platelet (10^9^/L)	270 [148-602]	268 [148-538]	270 [148-602]	0.738
CRP (mg/L)	4.1 [1.9-69.4]	4.4 [1.9-56]	4 [3-69.4]	0.948
Albumin (g/L)	4 [2.9-5.3]	4.1[2.9-5.1]	3.95 [2.9-5.3]	0.762
Hemoglobin (g/L)	11.9 [8,77-15,3]	12,3[9,57-15,3]	11,52 [8,77-15,3]	**0.012**
HCT (%)	36.4 [26.3-47.8]	36.8 [28.7-46]	34.65 [26.3-47.8]	**0.025**
MCV (fL)	79.3 [62.4-92.2]	77.75 [62.4-89.6]	79.3 [67.6-92.2]	**0.049**

Mann-Whitney U test; CEA, Carcinoembryonic antigen; WBC, White blood cell; CRP, C-reactive protein; HCT, Hematocrit; MCV, Mean corpuscular volume; MLN, Metastatic lymph node. P<0.05 statistically significant.

Most of patients underwent an open surgery (73.3%) and 57.43% had an epidural anesthesia. The mean duration of operation was 215.4 minutes and was not different between groups. 30.4% of the patients with MLN diameter ≥1.05 cm and 9.1% of the patients with MLN diameter <1.05 cm were given an intraoperative ES and this difference was found to be statistically significant (p=0.006). The distribution of Clavien-Dindo grade and postoperative ES need did not differ among the two groups. The patients with MLN diameter ≥1.05 cm were hospitalized longer than with MLN diameter <1.05 cm (median 9-day *vs.* 8-day, p=0.0294). The mortality rate was significantly higher in the group with MLN diameter ≥1.05 cm. (p=0.001) (**Table 7**).

**Table 7:** Intraoperative and postoperative findings.

Parameter		Total N = 101	MLN < 1.05cm (N = 55)	MLN ≥1.05cm (N = 46)	P value
Epidural Anesthesia, N (%)		58 (57.43)	32 (58.2)	26 (56.5)	0.867^a^
Operation Duration (minute) Mean ± SD		215.4±35.42	217±32.36	213.48±39.06	0.410^b^
Intraoperative ES, N (%)		19 (18.81)	5 (9.1)	14 (30.4)	**0.006** ^c^
Postoperative ES, N (%)	0	56 (55.45)	34 (61.8)	22 (47.8)	0.576^a^
	1	16 (15.84)	8 (14.5)	8 (17.4)	
	2	26 (25.74)	12 (21.8)	14 (30.4)	
	3	2 (1.98)	1 (1.8)	1 (2.2)	
	4	1 (0.99)	0 (0)	1 (2.2)	
Hospitalization Duration (day) Median [Range]		8 [6-49]	8 [6-16]	9 [6-48]	**0.0294** ^d^
Clavien-Dindo grade	0	34 (33.66)	20 (36.4)	14 (30.4)	0.455^a^
	1	19 (18.81)	11(20)	8 (17.4)	
	2	43 (42.57)	20 (36.4)	23 (50)	
	3	5 (4.95)	4 (7.3)	1 (2.2)	
Mortality, N (%)	Alive	69 (68.32)	51 (92.7)	18 (39.1)	**0.001** ^c^
	Deceased	32 (31.68)	4 (7.3)	28 (60.9)	

^a^Chi-Square test; ^b^Student's t-test; ^c^Fisher-Freeman-Halton test; ^d^Mann-Whitney U test; MLN, Metastatic lymph node; ES, Erythrocyte suspension; OS, Overall survival. P<0.05 statistically significant.

According to the pathological findings (**Table 8**), the median number of harvested LN was 22 and the median number of MLN was 4. The mean maximum diameter of the tumors was 6.49 cm, and most of them were in T3 stage (66.34%), N2 stage (50.5%) and nuclear stage 2 (67.33%). Lymphovascular invasion was present in 58.42% and perineural invasion was present in 41.58% of all tumors. T stage, N stage, nuclear grade of tumor, presence of lymphovascular and perineural invasion and total number of harvested LN did not differ among the two groups. However, the total number of MLN was higher in the patients with MLN diameter ≥ 1.05 cm (median 4) than those with MLN diameter < 1.05 cm (median 3) (p=0.049). The diameter of maximum tumor size was also considerably larger in patients with MLN diameter ≥ 1.05 cm (median 7.35 cm) than those with diameter < 1.05 cm (median 5.2 cm) (p=0.0001).

**Table 8:** Histopathological findings.

Parameter		Total N = 101	MLN < 1.05cm (N = 55)	MLN ≥ 1.05 cm (N = 46)	P value
		Mean ± SD Median [Range]			
Total number of LN		23.4±9.09 22 [11-46]	23.35±8.71 22 [11-46]	23.46±9.61 20 [12-45]	0.840^a^
Number of MLN		4.32±2.84 4 [1-17]	3.43±1.65 3 [1-8]	4.89±3.41 4 [1-17]	**0.049** ^a^
Maximum Diameter of Tumor (cm)		6.49±1.75 6.2[3.8-13.5]	5.42±0.9 5.2 [3.8-8.3]	7.76±1.66 7.35 [4.8-13.5]	**0.001** ^a^
Parameter		N (%)			
T stage	1	1 (0.99)	0 (0)	1 (2.2)	0.553^b^
	2	7 (6.93)	5 (9.1)	2 (4.4)	
	3	67 (66.34)	35 (63.6)	32 (69.6)	
	4	26 (25.74)	15 (27.3)	11 (23.9)	
N stage	1	50 (49.50)	30 (54.5)	20 (43.5)	0.268^b^
	2	51 (50.50)	25 (45.5)	26 (56.5)	
Lymphovascular invasion		59 (58.42)	35 (63.6)	24 (52.2)	0.244^b^
Perineural invasion		42 (41.58)	20 (36.4)	22 (47.8)	0.244^b^
Nuclear Grade	1	10 (9.90)	7 (12.7)	3 (6.5)	0.501^c^
	2	68 (67.33)	37 (67.3)	31 (67.4)	
	3	23 (22.77)	11 (23.9)	12 (26.1)	

^a^Student's t-test; ^b^Chi-Square test; ^c^Fisher-Freeman-Halton test; LN, Lymph node; MLN, Metastatic lymph node. P<0.05 statistically significant.

## Discussion

Our study results indicated that the evaluation of the largest MLN size *via* the histopathological examination can provide valuable information in CRC patients with metastatic lymph nodes. The main aim of this study was to examine the clinical significance of the largest MLN in terms of postoperative outcomes and its predictive value in mortality of patients with stage III CRC.

Lymph node (LN) metastasis in CRC is a prognostic factor, determines the disease stage and guides the treatment (4). Many studies showed the effect of MLN number and MLN ratio on survival (7). Some studies reported that MLN size is effective in determining the prognosis in patients with gastrointestinal malignancies, including gastric and esophageal cancer, and provides support for classification systems. In gastric cancer, larger MLN size was associated with poor prognosis and MLN size was important in overall and disease-free survival (DFS) rates. Similarly, the importance of metastatic LN size in CRC evaluated in a limited number of studies. In 2005, Dhar et al. measured the long diameter of 107 CRC patients’ MLN and reported that the overall survival of 69 patients with MLN size ≤ 9 mm was 63.5% and 38 patients with MLN size ≥ 10 mm was 42.5% (13). In 2022, Maeda et al. divided 209 patients who underwent curative colectomy for pathological stage III colon cancer, into four groups based on the short-axis diameter of the largest MLN<5 mm, ≥5 mm and <10 mm, ≥10 mm and <15 mm, ≥15 mm. There were no significant differences in OS rates between the groups. But, they found that the 5-year recurrence-free survival (RFS) rates of groups were 82.3%, 74.6%, 74.5% and 60.7%, respectively. MLN diameter ≥15 mm reported to be associated with significantly worse RFS in multivariate analysis (14). Survival for both colon and rectal cancer had improved over the years. 5-year survival increased from 53% to 62% and from 51% to 65% for all colon and rectal cancer stages, respectively (3). Consistent with the literature, our study had a 68.32% survival rate for all stage III CRC patients. However, a significant difference was found between the groups formed by considering the MLN diameter. Patients with MLN diameter ≥ 1.05 cm had a survival rate of 39.1% and OS of 52,57 months, whereas patients with an MLN diameter of <1.05 cm had a survival rate of 92.7% and OS of 75,29 months.

Tumor size is an independent factor for survival in CRC (15). Lymph node size is not a reliable indicator for lymph node metastasis (16). Metastatic lymph nodes can be seen in any size (17). Luo at al reported nonlinear correlation between tumor size and lymph node metastasis (18). According to the Dhar et al. study there was a significant correlation between MLN size and MLN number in CRC. Also, they indicated that the prevalence of MLN size≥ 10 mm increased with increasing depth of tumor penetration (13). Maeda et al. also showed that the median tumor size, advanced T and N stage status, and the number of MLNs were higher in patients with larger MLN diameter (14). In our study, the maximum size of tumor was larger and number of MLNs were higher among the patients with MLN diameter ≥ 1.05 cm than those of patients with diameter < 1.05 cm. However, T stage, N stage, lymphovascular/perineural invasion status and nuclear grades did not differ among the two groups. Therefore, histopathological measurement of MLN size may contribute to predicting the prognosis.

The blood biomarkers easily obtained from a preoperative routine blood test are associated with the prognosis of colorectal cancers. The immune cell counts, inflammatory and coagulation parameters and their ratios were examined for a prognostic factor of cancer. In a recent study, the neutrophil-lymphocyte ratio was proved to be an independent prognostic factor for disease-free survival in patients with non-metastatic colon cancer (19). In another study, Zhang at al. showed that pre-operative lymphocyte count was an independent prognostic factor and pre-operative high lymphocyte count was significantly associated with better prognosis of rectal cancer patients (20). In a study, in which clinical laboratory and morphological factors were evaluated in colon cancer, hematocrit value between 16.7% and 31% was found to be significantly correlated with pT > 2 (21). In our study, blood product was needed in the perioperative period in the patient group with MLN diameter ≥ 1.05 cm, worse survival, lower preoperative hemoglobin level and HCT values. In terms of MCV, Nagai et al. found that patients with MCV <80 fL superior to patients with MCV ≥80 fL for DFS and reported that MCV was a prognostic factor for DFS in CRC (22). Our study is in the same direction with the literature in terms of lymphocyte count, hemoglobin level, hematocrit ratio and MCV value.

Longer length-of-hospital stay and emergency admissions after colorectal surgery are not uncommon. According to Kelly et al., one in four patients remained in the hospital for at least 25 days after colorectal resection (23). In a study, age ≥ 76 years, CCS ≥ 2, total mesorectal excision (TME) and laparoscopic conversion were significantly associated with a prolonged hospital stay (24). Major complications require longer hospital stays. Postoperative complications adversely affect CRC survival (25). In our study, hospital stay was longer in patients with larger MLN size. However, we did not detect a relationship between the largest MLN size and the presence of major complications.

Limitations of the study are its retrospective single-center design and a relatively limited number of patients. Another limitation relates to our analysis of disease free survival. Since recurrence data was not set as one of the endpoints, these data had not been assessed systematically and were incomplete. However, there were a limited number of previous studies in this area. In contrast to Dhars’ and Maedas’ studies, not using categorical cut-off and preventing stage bias by including a limited pathological stage group are the strengths of our study. This paper expresses a different perspective on the relationship between postoperative complications and the largest MLN size.

## Conclusion

Our study results indicated that the largest MLN size was an independent risk factor for mortality and a cut-off value of 1.05 cm in MLN size had prognostic value in surgically treated stage III CRC patients. Therefore, the largest MLN size may be a proper alternative factor to number of MLNs for predicting the survival in CRC patients. In the light of these results, a review of N-stage subgroups of TNM staging may be considered. Further multicenter, large-scale studies are required to confirm our study results.

## Data availability statement

The raw data supporting the conclusions of this article will be made available by the authors, without undue reservation.

## Ethics statement

This study was approved by the ethical committee of Koş uyolu High Specialization Education and Research Hospital (Date: 10th Nov 2020, Issue number: 2020/12/382). Written informed consent for participation was not required for this study in accordance with the national legislation and the institutional requirements.

## Author contributions

SO and SG contributed to conception and design of the study. OU and AS organized the database. SO and SG performed the statistical analysis. EP and MD wrote the first draft of the manuscript. SO, SG, OG, and OB wrote sections of the manuscript. All authors contributed to manuscript revision, read, and approved the submitted version.
